# Targeting the Aryl Hydrocarbon Receptor Signaling Pathway in Breast Cancer Development

**DOI:** 10.3389/fimmu.2021.625346

**Published:** 2021-03-08

**Authors:** Christoph F. A. Vogel, Gwendal Lazennec, Sarah Y. Kado, Carla Dahlem, Yi He, Alejandro Castaneda, Yasuhiro Ishihara, Christian Vogeley, Andrea Rossi, Thomas Haarmann-Stemmann, Juliann Jugan, Hidetoshi Mori, Alexander D. Borowsky, Michele A. La Merrill, Colleen Sweeney

**Affiliations:** ^1^Department of Environmental Toxicology, University of California, Davis, Davis, CA, United States; ^2^Center for Health and the Environment, University of California, Davis, Davis, CA, United States; ^3^CNRS, SYS2DIAG, ALCEDIAG, Montpellier, France; ^4^Graduate School of Integrated Sciences for Life, Hiroshima University, Hiroshima, Japan; ^5^Leibniz Research Institute for Environmental Medicine, Düsseldorf, Germany; ^6^Center for Comparative Medicine, University of California, Davis, Davis, CA, United States; ^7^Department of Biochemistry and Molecular Medicine, School of Medicine, University of California, Davis, Davis, CA, United States

**Keywords:** AhR, AhRR, carcinogenicity, breast cancer, C/EBPβ, cyclooxygenase 2, inflammation

## Abstract

Activation of the aryl hydrocarbon receptor (AhR) through environmental exposure to known human carcinogens including dioxins can lead to the promotion of breast cancer. While the repressor protein of the AhR (AhRR) blocks the canonical AhR pathway, the function of AhRR in the development of breast cancer is not well-known. In the current study we examined the impact of suppressing AhR activity using its dedicated repressor protein AhRR. AhRR is a putative tumor suppressor and is silenced in several cancer types, including breast, where its loss correlates with shorter patient survival. Using the AhRR transgenic mouse, we demonstrate that AhRR overexpression opposes AhR-driven and inflammation-induced growth of mammary tumors in two different murine models of breast cancer. These include a syngeneic model using E0771 mammary tumor cells as well as the Polyoma Middle T antigen (PyMT) transgenic model. Further AhRR overexpression or knockout of AhR in human breast cancer cells enhanced apoptosis induced by chemotherapeutics and inhibited the growth of mouse mammary tumor cells. This study provides the first *in vivo* evidence that AhRR suppresses mammary tumor development and suggests that strategies which lead to its functional restoration and expression may have therapeutic benefit.

## Introduction

Environmental exposure to toxicants including dioxins and many other dioxin-like compounds (DLC) which bind to the aryl hydrocarbon receptor (AhR) and activate the AhR signaling pathway, is associated with the development of malignancies including breast cancer (1–4). Indeed, due in part to the extensive mechanistic information indicating that the DLCs act through a mechanism involving the AhR the International Agency for Research on Carcinogens named DLCs as “carcinogenic to humans” (Group 1) (5). The activated AhR pathway results in changes of the expression profile of cytokines and immune modulatory enzymes which may contribute to the carcinogenic effects of AhR-activating toxicants (3). The repressor protein of the AhR (AhRR) has been found to suppress the canonical AhR signaling pathway as well as the activation of inflammatory cytokines (6, 7). Moreover, reports have shown that the AhRR suppresses growth of tumor cells including breast cancer cells *in vitro* (8). Support for the premise comes from a recent report, showing that breast cancer patients who had low AhRR expression also had shorter metastasis-free survival and identified AhRR as an independent prognostic factor (9). Literature also indicates that the AhR regulates normal development of the mammary gland (10–12) revealing this tissue as a sensitive target of environmental pollutants containing AhR activating chemicals.

The finding of overexpressed AhR in mammary cancer in rats (13) raised the question of whether AhR is involved in breast cancer progression. Indeed, several *in vitro* studies demonstrated the contribution of AhR to carcinogenic progression (14). For instance, Brooks and Eltom (15) showed that overexpression of AhR in human mammary epithelial cells led to cellular transformation and epithelial to mesenchymal transition (EMT). Work from our group revealed that chronic exposure of human breast MCF10AT1 and MCF-7 cells to estradiol (E_2_) resulted in AhR overexpression and downregulation of estrogen receptor alpha (ERα) and progesterone receptor (16, 17). Both cell lines exhibited increased proliferation, matrigel invasion, and apoptosis resistance compared to control cells. More recently, we and other groups found that the AhR is frequently overexpressed in human breast cancer, particularly ER-negative breast cancer (9, 18, 19). AhR overexpression in this setting is closely associated with elevated expression of the NF-κB subunit RelB and the inflammatory markers IL-8 (CXCL1 in mouse) and COX-2 (19). Interestingly, COX-2 and chemokines such as CXCL1, CXCL5, and the chemokine receptor CXCR2 have been identified as critical genes that mediate breast cancer metastasis to lung, lymph nodes, and bone (20–22). A recent genome wide analysis of AhR and AhRR binding found a significant overlap in sequences binding both proteins, suggesting that AhRR most likely functions as a tumor suppressor by opposing AhR-driven gene expression (23).

Despite dysregulation of the AhR/AhRR axis in breast cancer, relatively little is known about the function of AhRR *in vivo* (6, 24). In the current study we have examined several AhR-driven outcomes, to determine whether AhRR functionally opposes AhR and is able to suppress the development of mammary tumors. Using our previously characterized AhRR transgenic mouse, we demonstrate that AhRR overexpression restricts the growth of both E0771 mammary tumor cells and mammary tumors in the Polyoma Middle T antigen (PyMT) model of mammary tumorigenesis. Furthermore, the tumor suppressive function of AhRR was confirmed in mouse PyMT-derived mammary tumor cells and human breast cancer cell lines indicating that AhRR inhibits cell proliferation and AhR-mediated apoptosis resistance.

## Materials and Methods

### Reagents

Dimethyl sulfoxide (DMSO) was purchased from Sigma. [γ-^32^P]ATP (6,000 Ci/mmol) was provided by ICN Biochemicals, Inc. (Costa Mesa, CA, USA). 2,3,7,8-tetrachlorodibenzo-p-dioxin (TCDD) (>99% purity) was originally obtained from Dow Chemical Co. (Midland, MI, USA). Other molecular biological reagents were purchased from Cayman Chemicals (Ann Arbor, MI, USA) and Applied Biosystems (Foster City, CA, USA).

### Cell Culture and Transfection Experiments

Mammary epithelial cells (UCD-PYMT) were isolated from the mammary tumor of a 26-week-old B6.FVBTg(MMTV PyVT)634Mul/LellJ (PyMT) hemizygous mouse (Jackson Laboratory, Bar Harbor, ME, USA) (25, 26) as described in Pénzváltó et al. (27). Briefly, the mammary tumor was washed twice in PBS (Invitrogen, Carlsbad, CA, USA) before it was mechanically dissociated and minced in a solution of serum-free DMEM:F12 (Invitrogen) with HEPES (Invitrogen), supplemented with 0.5 mg/ml Penicillin/Streptomycin (Invitrogen), 2% bovine serum albumin fraction V (Invitrogen), 5 μg/ml insulin (Sigma Aldrich, Saint Louis, MO), 10 ng/ml cholera toxin (Sigma Aldrich, USA), and 3 mg/ml collagenase (Worthington Biochemical Corp., Lakewood, NJ). The tissue was then digested with gentile agitation overnight at room temperature before differential centrifugation at 80× g for 1.5 min. The remaining cell pellet of UCD-PYMT cells was washed in DMEM:F12 (Invitrogen) and centrifuged at 80× g for 4 min before being cultured in Advanced DMEM/F12 culture medium (Gibco, Thermo Fisher scientific Inc., Waltham, MA, USA) supplemented with 5% FBS, 1% ITS Premix (Corning, Concord, NC), 0.5 mg/ml Penicillin/Streptomycin (Invitrogen), and 1% GlutaMax (Gibco) as described (25, 26). UCD-PYMT epithelial origin was further confirmed by 100% E-cadherin positive staining of cells (DAPI positive) at 24 h and after 5 days in culture (Supplementary Figure 1).

UCD-PYMT were transiently transfected with a cDNA mouse AhRR expression plasmid or an A-C/EBP vector that produce dominant-negative proteins that specifically inhibit the DNA binding of the C/EBP members kindly provided by Charles Vinson (NCI, Bethesda, MD, USA). Transient transfection was performed using jetPEI (PolyTransfection; Qbiogene, Irvine, CA, USA), according to the manufacturer's instructions. The transfection was allowed to proceed for 16 h, and cells were treated with 1 nM TCDD or 0.1% DMSO (control) for 24 h before induction of apoptosis or treatment with TCDD for RNA expression analysis. For DRE luciferase reporter assay UCD-PYMT cells were transiently transfected with a DRE reporter plasmid. After 16 h cells were treated with 1 nM TCDD or 0.1% DMSO (control) for 4 h. Cells were lysed and luciferase activity was measured with the Luciferase Reporter Assay System (Promega Corp., Madison, WI, USA) using a luminometer (Berthold Lumat LB9501/16; Pittsburg, PA, USA). Relative light units were normalized to protein concentration using Bradford dye assay (Bio-Rad Laboratories, Inc., Hercules, CA, USA).

MDA-MB 231 and MCF-7 cells were cultured in DMEM plus 10% FCS. Cells were seeded in 12-well plates at 2 × 10^5^ cells/well. After 24 h cells were transfected with a rat AhRR expression plasmid, which was generously provided by Yoshio Inouye. Control cells were transfected with the empty vector. Apoptosis was induced with Etoposide (50 μM) and Doxorubicin (10 μM) (Cayman Chemical, Ann Arbor, MI, USA) and control cells received 0.1% DMSO vehicle.

### Generation of CRISPR/Cas9 AhR Mutants of MDA-MB 231 and MCF-7 Cells

A gRNA targeting AhR exon 2 (5′-AAGTCGGTCTCTATGCCGCTTGG-3′) was designed using the CRISPR design tool CHOPCHOP (http://chopchop.cbu.uib.no/) and cloned into a modified version of the PX458 plasmid available on Addgene (48138). The resulting bicistronic vector encoded the gRNA and the Cas9 nuclease. gRNA activity and efficiency were assessed using High Resolution Melt Analysis (HRMA) (28) using the following primers: fw 5′-GCCAATCCCAGCTGAAGG-3′ rv 5′-TAGCCAAACGGTCCAACTCT-3′ and a MyGo PRO real time PCR (IT-IS Life Science Ltd). MDA-MB 231 and MCF-7 cells were transfected with nuclease plasmids in antibiotic-free medium in a 12-well plate using FuGENE HD (Roche) according to the manufacturer's protocol. After 48 h cells were sorted (FACS) and plated as single cells in a 96-well plate and duplicated after a week. Clones were lysed in Proteinase-K and genotyped using high-resolution melt analysis and SANGER sequencing. AhR knockout in MCF-7 and MDA-MB 231 cells was confirmed in Western blot analysis (**Figures 6E,F**).

### Mice and Treatment

The mice (C57BL/6J background) used in our experiments include B6.FVB-Tg(MMTV-PyVT)634Mul/LellJ hemizygous mice transgenic for the PyMT oncogene driven by the mouse mammary tumor virus long terminal repeat (MMTV-LTR) (29, 30). C57BL/6J wild type (wt) and PyMT mice were purchased from the Jackson Laboratory (Sacramento, CA, USA). PyMT mice were crossed with AhRR Tg mice to generate PyMT/AhRR+ mice double transgenic for PyMT and the mouse AhR Repressor (AhRR). AhRR Tg and PyMT mice were genotyped using the DNA/RNA Shield reagent (Zymo Research, Irvine, CA, USA) for nucleic acids isolation. Mice were housed in a selective pathogen-free facility at UC Davis. Mice were maintained on a 12:12 h light/dark cycle and had free access to water and food according to the guidelines set by the University of California. The protocol for animal care and use was approved and completed by the Institutional Animal Care and Use Committee (IACUC) on February 06, 2020 at the University of California, Davis (#21564). This project was conducted in accordance with the ILAR guide for the care and use of laboratory animals, and the UC Davis Animal Welfare Assurance on file with the US Public Health Service.

To address the tumor-suppressive action of AhRR *in vivo*, we used a syngeneic murine breast cancer model to evaluate in tumor susceptibility in wt and AhRR Tg mice. To create tumors, we used an orthotopic xenograft tumor model by subcutaneous (s.c.) injection of E0771 breast cancer cells according to (31). The E0771 cell line is a spontaneously developing medullary breast adenocarcinoma from C57BL/6 mice (30). The cultured E0771 tumor cell suspension was resuspended in PBS to obtain the desired concentration of 5.0 × 10^6^ cells/mL. A 1 mL syringe affixed with a 23-G needle was loaded with 0.1 mL of the E0771 tumor cell suspension (500,000 cells). For control, 0.1 mL PBS alone was injected. E0771 cells were injected subcutaneously into the fourth inguinal mammary gland of wt and AhRR Tg mice (10 weeks old, 10 female mice in each group). Twenty-four hours after injection of E0771 cells, mice were treated with vehicle (corn oil or PBS) or TCDD (10 μg/kg bw) in order to test possible enhancing effects of TCDD on tumor growth of E0771 breast cancer cells in wt and AhRR Tg mice. TCDD was administered *via* intraperitoneal (i.p.) injection. Each mouse was palpated three times a week at the injection site and the tumor size was measured using a slide microcaliper for 18 days post-injection. These data were used to determine the tumor volume by employing the following formula V = (L*W*H)/2.

Virgin mammary glands and lungs from 5-month-old PyMT/wt and PyMT/AhRR^+^ transgenic female mice were prepared at necropsy for histology. Whole mounts were spread on slides, fixed and stained with hematoxylin in order to elucidate ductal structure as described (32).

### Electrophoretic Mobility Shift Assay (EMSA)

Nuclear extracts were isolated from UCD-PYMT as described previously (33). UCD-PYMT were treated with TCDD for 90 min and harvested in ice cold Dulbecco's PBS. The DNA/protein binding reactions were carried out in a total volume of 15 μL containing 10 μg of nuclear protein, 60,000 cpm of double-stranded C/EBP consensus oligonucleotide (5′-TGCAGATTGCGCAATCTGCA-3′) plus 1 μg of poly(dI·dC). The samples were incubated at room temperature for 20 min. Competition experiments were performed in the presence of a 100-fold molar excess of unlabeled oligo. Protein-DNA complexes were resolved on a non-denaturating polyacrylamide gel and visualized by exposure of the dried gels to x-ray films. Protein-DNA complexes were quantified using ChemImager™ 4400 (Alpha Innotech Corp.).

### RNA Isolation and Real-Time PCR

Total RNA was isolated from cells using a Quick-RNA Mini prep isolation kit (Zymo Research), and cDNA synthesis was performed as described (33) using a cDNA synthesis kit Applied Biosystems (Foster City, CA, USA). Detection of β-actin and differentially expressed target genes was performed with a LightCycler LC480 Instrument (Roche Diagnostics, Indianapolis, IN, USA) using the Fast SYBR Green Master Mix (Applied Biosystems) according to the manufacturer's instructions. The primers for each gene were designed on the basis of the respective cDNA or mRNA sequences using OLIGO primer analysis software provided by Steve Rozen and the Whitehead Institute/Massachusetts Institute of Technology Center for Genome Research so that the targets were 100–200 bp in length. PCR amplification was carried as described (33). To confirm the amplification specificity, the PCR products were subjected to melting curve analysis.

### Western Blotting

Proteins from mouse tissue samples were isolated and prepared for Western blot as described (7). Cells were collected and lysed with radioimmunoprecipitation assay buffer and equal amounts of protein were loaded, separated *via* SDS-PAGE and transferred onto polyvinylidene difluoride membranes. The blocked membranes were incubated with the specific antibodies. The antibodies against actin and human AhR were purchased from Cell Signaling Technologies (Danvers, MA, USA), while the purified rabbit anti-AhRR antibody was purchased from Novoprotein (Fremont CA, USA) and mouse AhR purchased from Enzo (Farmingdale, NY, USA). Bands were visualized using peroxide substrates (SuperSignal West Pico, ThermoFisher Scientific, USA) after incubation with a peroxide-conjugated antibody. The band intensity was quantified using ChemImager™ 4400 (Alpha Innotech Corp.).

### Cell Growth

UCD-PYMT cells and AhRR or A-C/EBP transfected UCD-PYMT cells were seeded at a density of 2 × 10^4^ per mL of growth medium in 48-well plates and were incubated overnight. At 24, 48, 72, and 96 h, 20 μL (5 g/L) of MTT (3-(4,5-dimethyl-2-thiazolyl)-2,5-diphenyl-2-*H*-tetrazolium bromide) reagent was added to the designated wells. After a 4 h incubation, the MTT formazan precipitate was dissolved in DMSO and the absorbance was determined at 490 nm using a plate reader (Berthold, USA).

### Apoptosis Assay

UCD-PYMT cells (5 × 10^5^ cells) were seeded in a 6 cm dish and exposed to TCDD for 24 h prior to apoptosis induced by Etoposide and Doxorubicin and detected by Annexin V staining as described previously (34). The detection of the phosphorylated form of variant histone H2AX (γ-H2AX), which occurs specifically at sites of DNA double-strand breaks was used to determine apoptotic cells *via* flow cytometry in MDA-MB 231 and MCF-7 cells. Cells were seeded in 12-well plates at 2 × 10^5^ cells/well. For FACS analyses the supernatant was collected and the cell layer was washed with PBS and trypsinized. Trypsinized cells were collected and transferred to the respective supernatant. Cells were centrifuged at 300 g for 5 min at room temperature, followed by washing with PBS. Pellets were resuspended in 300 μl fluorochrome solution containing 0.1% sodium citrate, 0.1% Triton X-100, 50 μg/ml propidium iodide and 25 ng APC anti- γH2AX (Ser139) antibody (BioLegend, San Diego, CA, USA). After incubation in the dark for 20 min, cells were analyzed by flow cytometry. The acquired data were analyzed using the FlowJo software package (Tree Star Inc., Ashland, OR, USA).

### Statistical Analysis

All experiments were repeated a minimum of three times, and data were expressed as mean ± S.D. Differences were considered significant at *p* < 0.05. A comparison of two groups was made with an unpaired, two-tailed Student's *t*-test. A comparison of multiple groups was made with analysis of variance followed by a Dunnett's or Tukey's test.

## Results

### Suppression of Tumor Growth in AhRR Tg Mice

**Figure 1:**
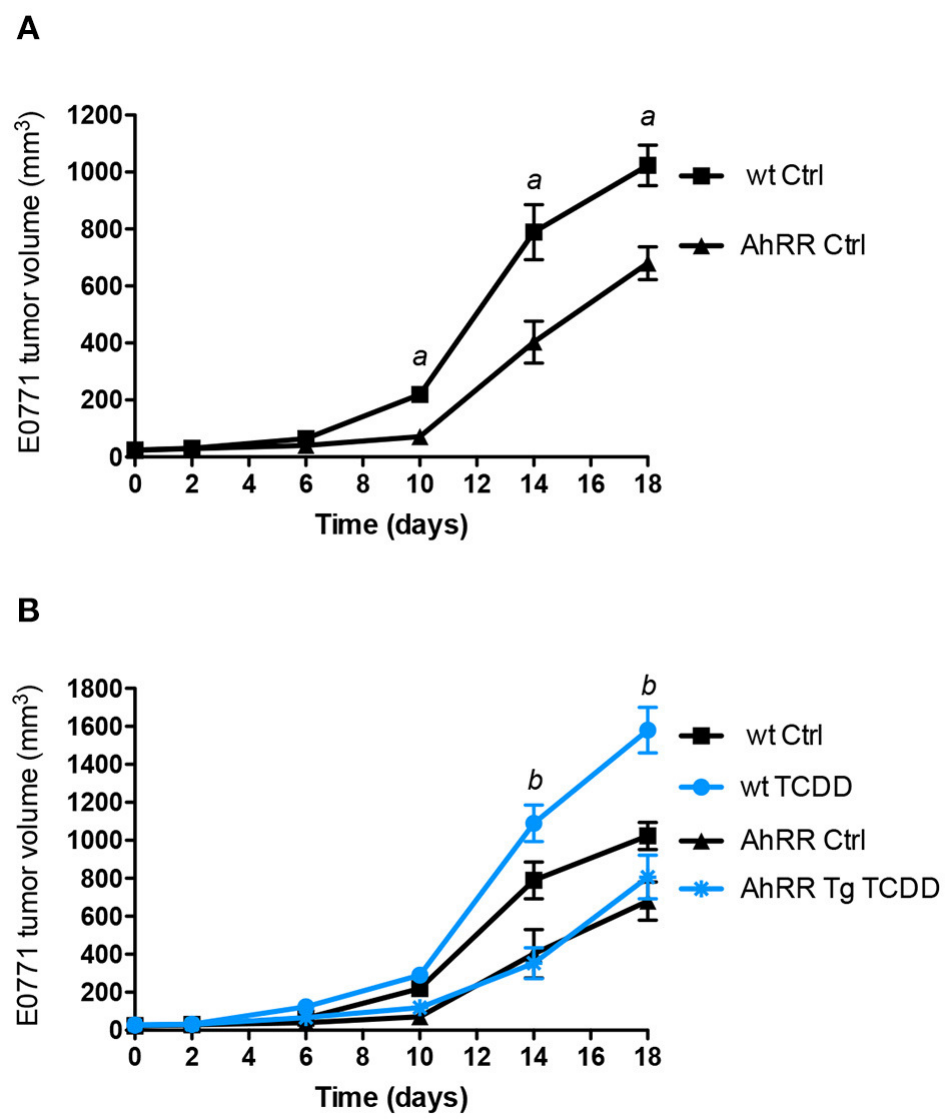
Suppressed tumor growth of E0771 tumor cells in AhRR Tg mice. **(A)** Tumor volume of 10-week-old control AhRR Tg mice and littermate wt mice (*n* = 8 for each group) following subcutaneous injection of 5.0 × 10^5^ E0771 breast cancer cells into the mammary fat pad. **(B)** After 24 h mice were i.p. injected with 10 μg/kg TCDD (blue lines). Mean ± SEM are shown and two-tailed Student's *t*-test was used. ^a^Significantly higher than AhRR Tg Ctrl mice, ^b^significantly higher than wt Ctrl or TCDD-treated AhRR Tg mice (*p* < 0.01).

To examine the tumor-suppressive action of AhRR *in vivo*, we compared growth of syngeneic E0771 mammary tumor cells in the mammary fat pad of wildtype (wt) B6 and AhRR Tg mice. The E0771 cell line is a spontaneously developing medullary breast adenocarcinoma derived from C57BL/6 mice and a model of triple negative breast cancer (TNBC). AhRR Tg mice were created and previously characterized by our group and exhibit overexpression of AhRR in all tissues examined (7). Results indicate a significantly suppressed growth of E0771 mammary tumor cells in AhRR Tg mice compared to wt mice (Figure 1A). Tumor growth was significantly enhanced after TCDD treatment only in wt mice while still suppressed in AhRR Tg mice (Figure 1B). TCDD is a prototypical ligand of AhR and the most toxic congener of dioxins. These data indicate that the overexpression of AhRR in the host environment is sufficient to suppress AhR-driven mammary tumor growth.

### AhRR Increases Tumor Latency and Decreases Tumor Incidence in the PyMT Model

**Figure 2:**
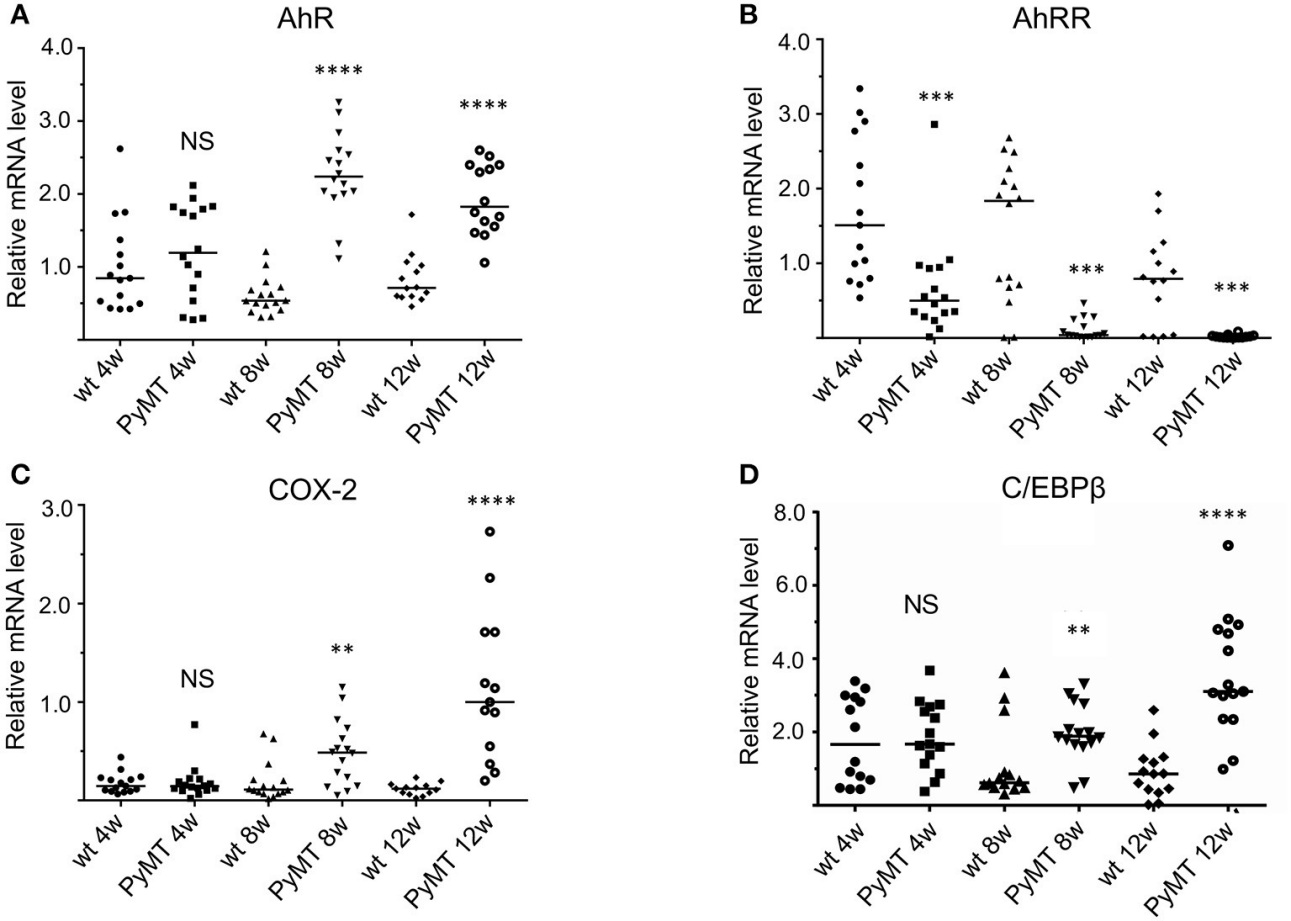
mRNA Expression of marker genes **(A)** AhR, **(B)** AhRR, **(C)** COX-2, and **(D)** C/EBPβ in mammary tissue during tumor development from 4 to 12 weeks in PyMT mice (*n* = 15) compared to wt mice (*n* = 15). NS, not significant; plot of single data points of 15 mice in each group are shown and two-tailed Student's *t*-test was used to test for statistical significance. Significantly different **p < 0.01, ***p < 0.001, ****p < 0.0001.

To further define the role of AhRR in mammary tumorigenesis, we chose the polyoma middle T antigen (PyMT) model, a widely used model of metastatic breast cancer. As shown in Figure 2, the PyMT model reflects expression changes observed in human breast cancer. Specifically, expression of AhR increases in mammary tissue during tumor progression with expression of AhR gene targets and inflammatory markers (e.g., COX-2 and C/EBPβ) increasing accordingly. In contrast, the expression of AhRR decreases, suggesting that the healthy “yin and yang” of AhR and AhRR is disrupted, favoring AhR signaling.

**Figure 3:**
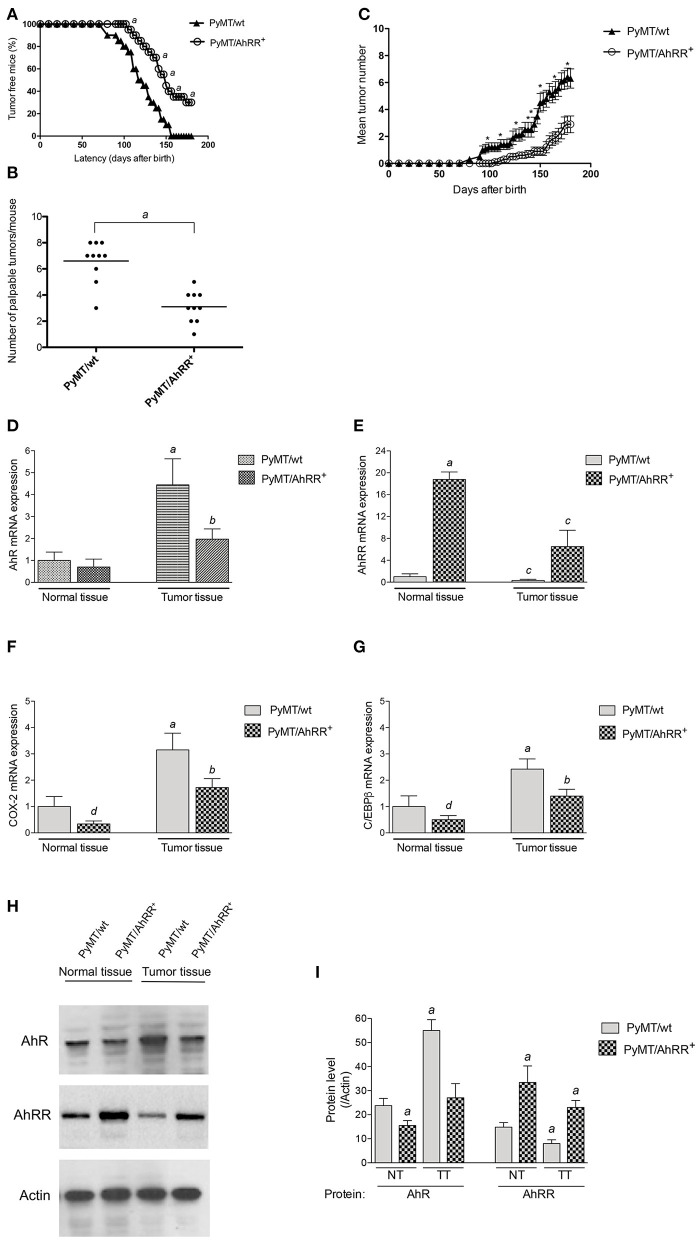
Overexpression of AhRR in PyMT mice extends mammary tumor latency and decreases tumor incidence. **(A)** Kinetics of palpable tumor onset in 10 PyMT/wt and 10 PyMT/AhRR^+^ mice. Values are shown as percentage of 10 mice. **(B)** AhRR overexpression decreases the number of palpable lesions detected at necropsy in PyMT/AhRR^+^ compared to PyMT/wt mice. Values of 10 mice per group are shown, ^a^significantly different from PyMT/wt, *P* ≤ 0.001. **(C)** Multiplicity of Mammary Tumors in PyMT/wt and PyMT/AhRR^+^ mice at the indicated time points are shown. Mean±SEM of are shown. *Statistically significant differences were tested by Student's *t*-test in tumor multiplicity, *P* ≤ 0.001. Expression of **(D)** AhR, **(E)** AhRR, **(F)** COX-2, and **(G)** C/EBPβ mRNA levels in normal and mammary tumor tissue of PyMT/wt and PyMT/AhRR^+^ mice. ^a^Significantly higher than normal mammary tissue of PyMT/wt mice, ^b^significantly lower than PyMT/wt tumor tissue, ^c^significantly lower than normal mammary tissue of PyMT/wt and PyMT/AhRR^+^ mice, ^d^significantly lower than PyMT/wt normal mammary tissue. Statistical significance was tested with two-way ANOVA test (*p* < 0.01). **(H)** Representative images of immunoblotting of AhR and AhRR in normal (NT) and mammary tumor tissue (TT). **(I)** The band intensity was measured, and the protein levels of AhR and AhRR were divided by those of Actin to calculate the relative protein levels. The values represent the mean ± SD (*n* = 3) and statistics of a Student's *t*-test are shown. ^a^Significantly different from PyMT/wt normal tissue (*p* < 0.01).

**Figure 4:**
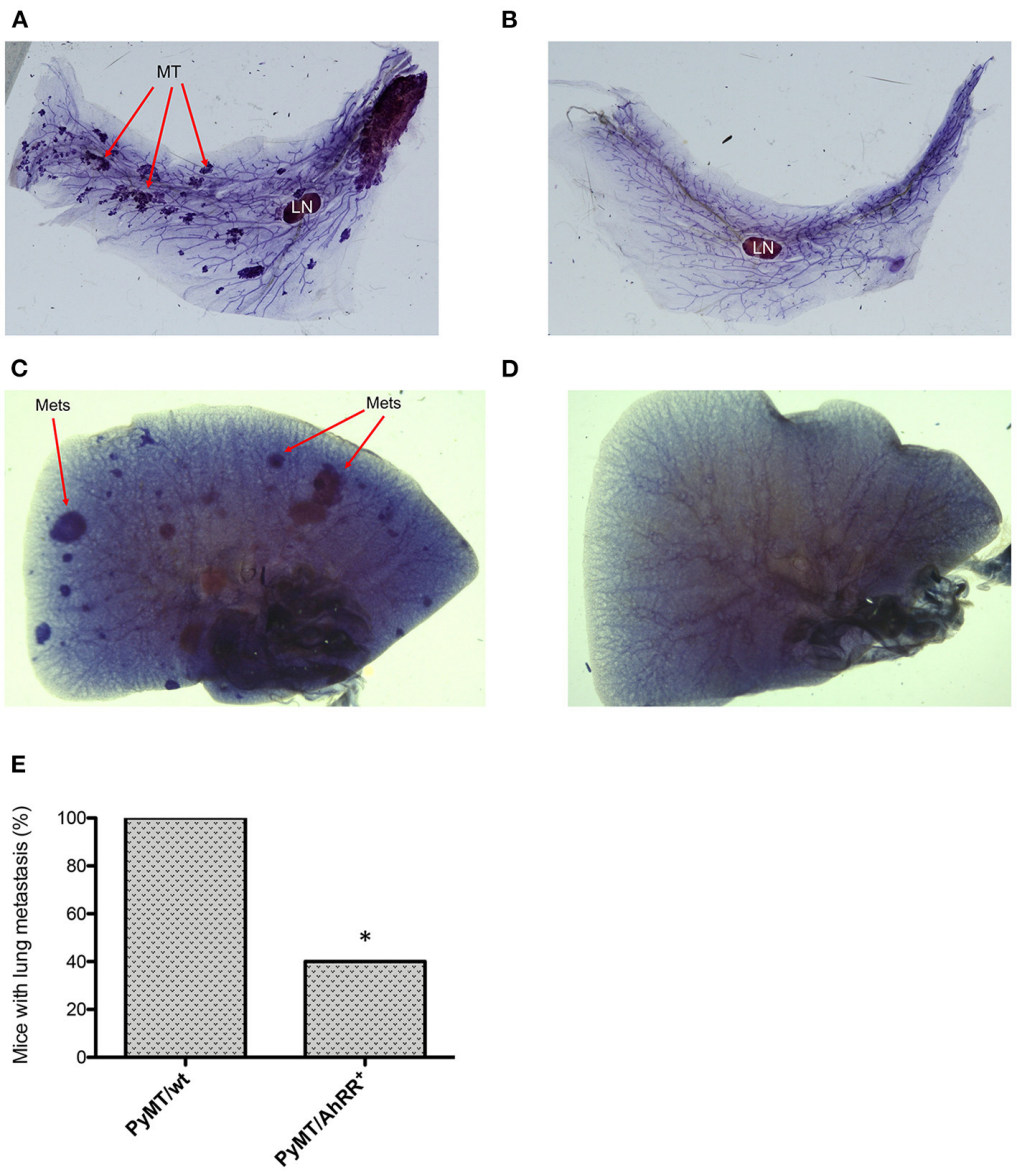
Whole-mounted mammary glands and lungs from PyMT/wt and PyMT/AhRR^+^ mice. Virgin mammary glands from 5-month-old **(A)** PyMT/wt and **(B)** PyMT/AhRR^+^ transgenic female mice. Whole-mounts were spread on slides, fixed and stained with hematoxylin in order to elucidate ductal structure. Mammary glands from 5-month-old PyMT/wt mice compared with a tumor free PyMT/AhRR^+^ transgenic female mice are shown. Arrows indicate MT (mammary tumors) and intramammary LN (lymph node). Overexpression of AhRR in PyMT mice suppressed lung metastasis in PyMT mice. Whole-mounted lungs from **(C)** PyMT/wt and **(D)** PyMT/AhRR^+^ mice at 5 months after birth. Whole mounts were spread on slides, fixed and stained with hematoxylin in order to elucidate metastatic lung tumors (Mets) as indicated. **(E)** Percentage of 10 PyMT/wt and PyMT/AhRR^+^ mice with lung metastasis at necropsy. Fisher's exact test was applied and value was statistically significant different, *P = 0.0108.

We next generated PyMT/AhRR^+^ mice and followed tumor growth over time. As shown in Figure 3, AhRR overexpression (PyMT/AhRR^+^) has a significant impact on tumor kinetics, increasing time to palpable tumor onset and decreasing incidence by the study censor date (Figure 3A). AhRR overexpression also decreased the number of palpable tumors at necropsy and reduced tumor multiplicity (Figures 3B,C). Expression analysis confirmed that AhR as well as COX-2 and C/EBPβ were suppressed in mammary tumors of PyMT/AhRR^+^ mice compared to PyMT/wt mice (Figures 3D–H). Representative whole mounts of mammary glands from 5 months old PyMT/wt and tumor free PyMT/AhRR^+^ mice are shown in Figures 4A,B. As expected, multi-focal mammary tumors are evident in PyMT/wt mice 5 months after birth. Furthermore, while metastatic colonies were evident in whole mounts of lungs from PyMT/wt mice (Figure 4C), PyMT/AhRR^+^ mice showed no evidence of lung metastasis 5 months after birth (Figure 4D). At necropsy 60% of PyMT/AhRR^+^ mice were devoid of lung metastasis, whereas all PyMT/wt mice developed metastatic foci in lung at necropsy (Figure 4E).

### AhRR Suppresses AhR-Induced Expression of Inflammatory Markers

**Figure 5:**
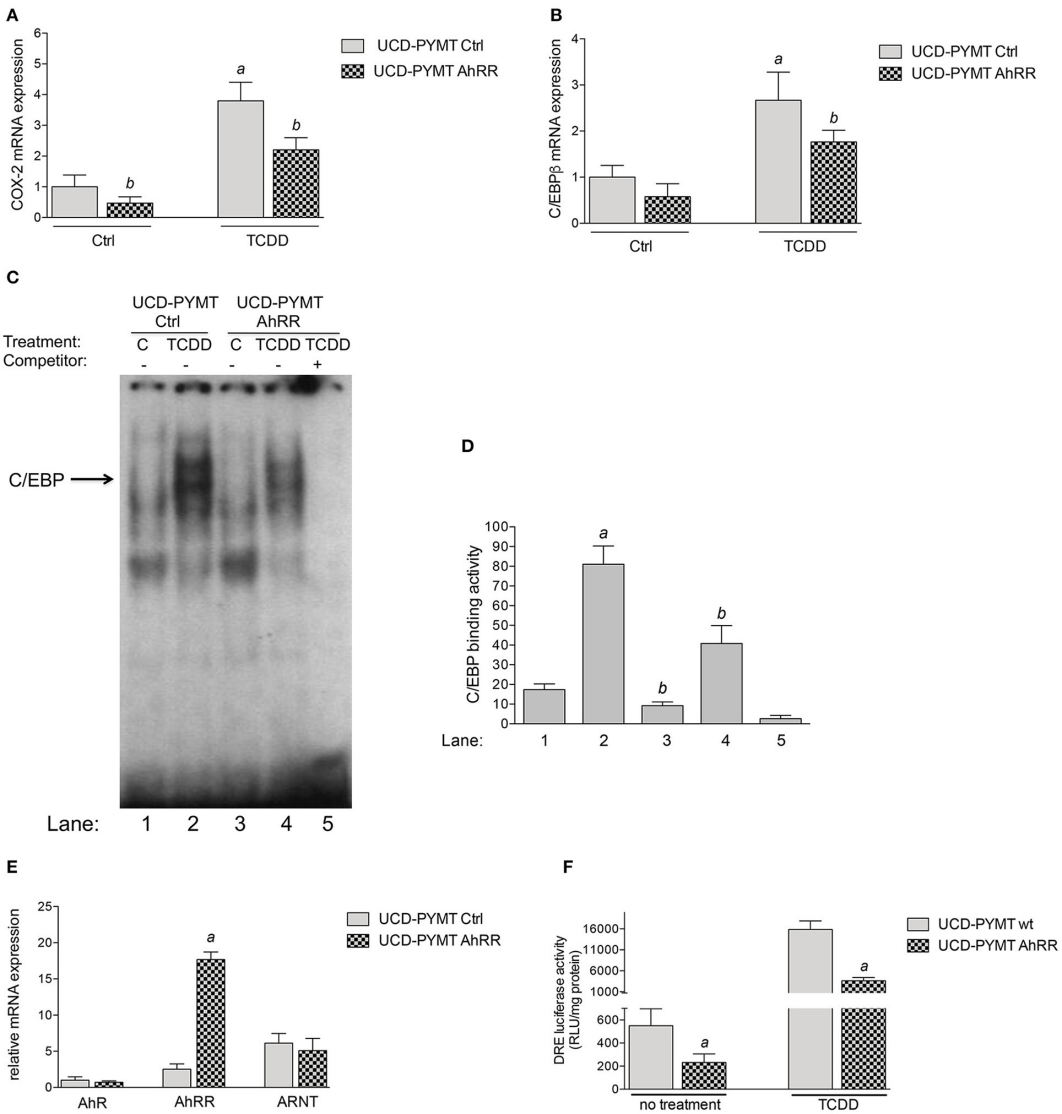
Effect of AhRR on the expression of COX-2, C/EBPβ and AhR activity in UCD-PYMT cells. UCD-PYMT cells were treated with TCDD (1 nM) for 24 h after transfection with an AhRR cDNA expression vector or an empty vector for 16 h before prior to treatment. mRNA expression levels of **(A)** COX-2 and **(B)** C/EBPβ were expressed as the ratio to that of GAPDH. Values are averages of duplicates from three different experiments. ^a^Significantly higher than control; ^b^significantly lower than UCD-PYMT Ctrl, *P* ≤ 0.01. **(C)** Repressed DNA binding activity to a C/EBP consensus element in AhRR transfected UCD-PYMT. UCD-PYMT were transfected with a control vector (lanes 1 and 2) and mouse AhRR cDNA expression plasmid (lanes 3 and 4) and treated with 1 nM TCDD (lanes 2 and 4). After 4 h nuclear proteins were extracted. For specificity a 200-fold molar excess of unlabeled probe was added as competitor (lane 5). **(D)** Densitometric evaluation of band intensities of the C/EBP DNA binding complexes. Band intensity of DNA binding complexes of nuclear proteins to C/EBP consensus element is shown as densitometry data. Numbers on the x-axes correspond to the lane numbers shown in **(D)**. Averages from three different experiments are shown as mean values ± SD. ^a^Significantly higher than control; ^b^significantly lower than UCD-PYMT Ctrl, Statistical significance was tested with a two-way ANOVA test, *P* ≤ 0.01. **(E)** Expression of AhR, AhRR, and ARNT in UCD-PYMT cells. UCD-PYMT cells were treated transfected with an AhRR cDNA expression vector or an empty vector for 16 h and mRNA expression was analyzed using qPCR. Relative expression levels are expressed as the ratio to that of GAPDH relative to the mRNA level of AhR in UCD-PYMT Ctrl. Values are averages of duplicates from three different experiments. ^a^Significantly higher than control, *P* ≤ 0.01. **(F)** Suppressed AhR activity in AhRR overexpressing UCD-PYMT cells. Cells were treated with TCDD (1 nM) for 4 h after co-transfection with a DRE-luciferase reporter plasmid and an AhRR cDNA expression vector or an empty vector for 16 h before prior to treatment. Values are given as mean ± SD of three independent experiments. ^a^Significantly lower than UCD-PYMT Ctrl, Student's *t*-test was used, *P* ≤ 0.01.

To further examine the effect of AhRR on the expression of COX-2 and C/EBPβ and to test mammary tumor cell-intrinsic effects of AhRR overexpression, we utilized UCD-PYMT cells, a mammary tumor cell line previously established from PyMT mice. UCD-PYMT cells were transfected with control plasmid or plasmid expressing AhRR and treated with 1 nM TCDD to engage AhR signaling. After 24 h, the expression of both C/EBPβ and COX-2 was induced by TCDD (Figures 5A,B). Notably, this induction was significantly restricted, for both C/EBPβ and COX-2 in UCD-PYMT overexpressing AhRR (Figure 5C). As reported earlier, the TCDD-mediated induction of COX-2 may involve the activation of PKA and DNA binding of C/EBPβ (35, 36). Therefore, DNA binding activity of C/EBPβ was determined utilizing EMSA with nuclear proteins prepared from control and AhRR-transfected UCD-PYMT cells. TCDD stimulated DNA binding to a C/EBP consensus element in both cases but this binding was significantly decreased in control and TCDD-treated AhRR overexpressing cells (Figures 5D,E). Furthermore, we found that AhRR reduced the basal as well as TCDD-induced activity of AhR in UCD-PYMT cells (Figure 5F) indicating the presence of endogenous ligands causing an increased constitutive level of AhR activity in these cells.

### AhRR Overexpression Inhibits Cell Growth and Sensitizes UCD-PYMT and MDA-MB 231 and MCF-7 Cells to Apoptosis Induced by Anti-cancer Drugs

**Figure 6:**
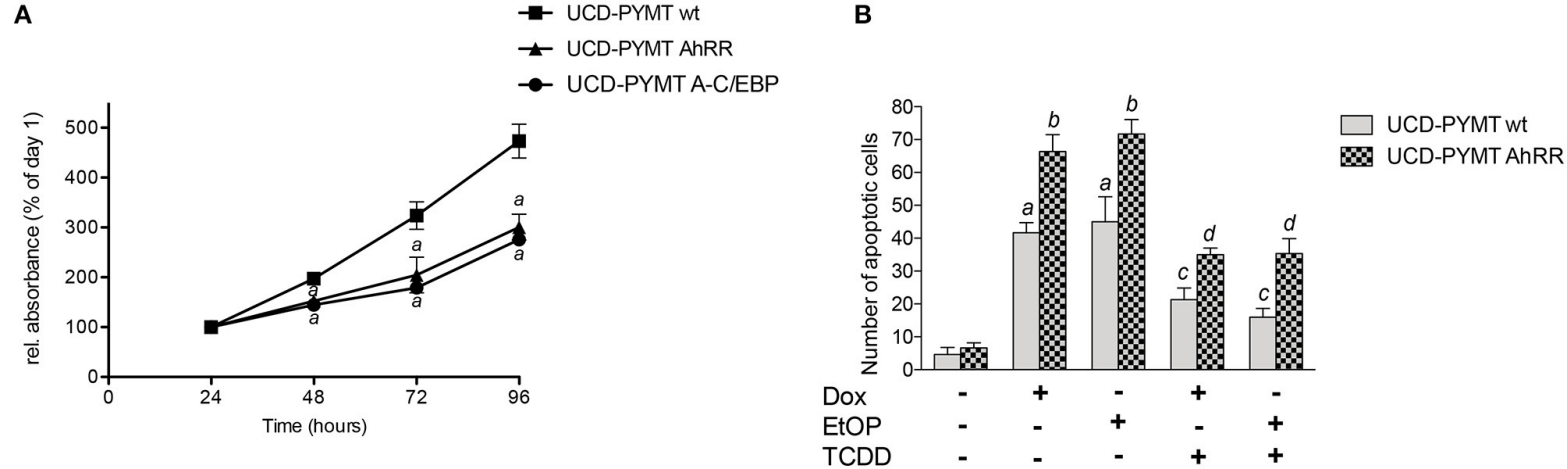
AhRR overexpression inhibits cell growth and enhances drug-induced apoptosis in UCD-PYMT. **(A)** The growth inhibitory effect of AhRR on UCD-PYMT cells. UCD-PYMT were transfected with an AhRR cDNA expression vector (UCD-PYMT AhRR) or an empty vector (UCD-PYMT wt). To test the role of C/EBPβ in cell proliferation cells were transfected with a C/EBP dominant negative expression plasmid (UCD-PYMT A-C/EBP). After transfection UCD-PYMT cells (2 × 10^4^/mL) were seeded in growth medium in 48-well plates. Culture medium was changed every 2 d. Cell proliferation rate was determined after 24–96 h by MTT assay. The results are the mean S.D. (*n* = 8) of the absorbance ratio on each day to the corresponding values on day 1. ^a^Significant lower compared to the values of UCD-PYMT Ctrl, Student's *t*-test was used *P* ≤ 0.01. **(B)** To test the effect of AhRR on apoptosis, UCD-PYMT were transfected with an AhRR cDNA expression vector (UCD-PYMT AhRR) or an empty vector (UCD-PYMT wt) for 16 h before cells were treated with TCDD (1 nM) for 1 h prior to treatment with Dox (5 μM) and EtOP (5 μM) for 24 h. Number of UCD-PYMT apoptotic cells was determined by Annexin V staining. Values are averages of duplicates from three different experiments. ^a^Significantly higher than control; ^b^significantly higher than UCD-PYMT wt; ^c^significantly lower than non-TCDD treated cells; ^d^significantly higher than TCDD-treated UCD-PYMT wt cells. Statistical significance was tested with a two-way ANOVA, *P* ≤ 0.01.

A hallmark of neoplastic development is deregulated cell proliferation and resistance to apoptosis. Previous reports have shown that AhRR inhibits cell growth and resistance to apoptotic signals in human breast epithelial and cancer cells (8, 17, 37). Here we tested the effect of AhRR on cell growth in UCD-PYMT cells after transfection with a mouse AhRR expression plasmid (Figure 6A). Cell proliferation rate was monitored from day 1 through day 4. The results show that AhRR significantly reduced the growth of UCD-PYMT cells compared to control cells (Figure 6A). In order to test if C/EBP binding plays a role in the inhibitory effect on cell growth we transfected cells with a vector expressing dominant negative proteins to block DNA binding of C/EBP proteins. The results show that A-C/EBP inhibits the growth of UCD-PYMT cells similar to overexpression of AhRR.

**Figure 7:**
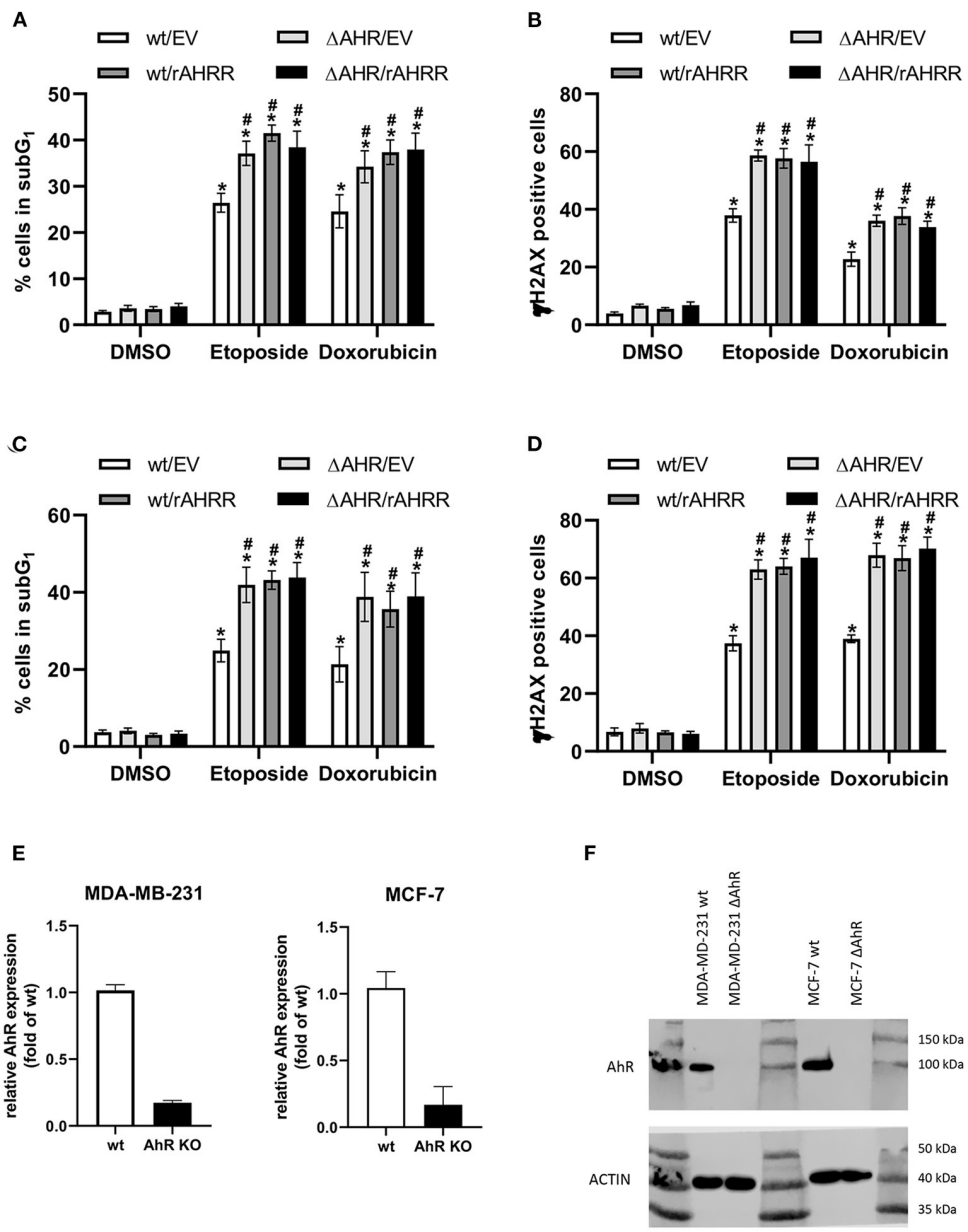
AhR deficiency and AhRR overexpression enhance drug-induced apoptosis and double-strand breaks (DSB) in human MDA-MB 231 and MCF-7 cells. Transiently AhRR overexpressing, AhR-knockout and control **(A,B)** MDA-MB 231 and **(C,D)** MCF-7 cells were treated with Dox and EtOP as indicated. After 48 h (MDA-MB 231) and 72 h (MCF-7), **(A,C)** apoptosis (percentage of cells in subG_1_) and **(B,D)** number of γH2AX-positive indicating DSB breast cancer cells were determined. Statistical significance was tested with a two-way ANOVA and multiplicity adjusted *P*-values were computed with Tukey's *post-hoc* test. *p < 0.05 compared to the respective DMSO treated sample. ^#^*p* < 0.05 compared to the respective wt/EV sample. **(E)** mRNA expression of AhR and **(F)** protein level of AhR in MDA-MB 231 and MCF-7 wt and AhR knockout cells.

Given our prior findings that AhR signaling mediates breast cancer cell resistance to apoptosis induced by UV radiation or anti-cancer drugs (34), we next examined the impact of AhRR expression on response of UCD-PYMT cells to doxorubicin (Dox), a DNA intercalating agent and etoposide (EtOP), an inhibitor of Topoisomerase-II. Treatment of UCD-PYMT cells with either Dox or EtOP led to a significant increase in apoptosis which was rescued in both cases by TCDD/AhR signaling (Figure 6B). More apoptosis was observed in AhRR overexpressing cells, for both Dox and EtOP. AhRR overexpression augmented apoptosis in response to both Dox and EtOP and mitigated the rescue provided by TCDD. This suggests that functional restoration of AhRR to breast cancer cells may be useful in addressing chemoresistance, a major driver of breast cancer mortality. Next, we investigated whether a modulation of AhR activity affects drug-induced apoptosis consistently in human triple negative MDA-MB 231 breast cancer cells (Figure 7A). Moreover, experiments with luminal ER-positive MCF-7 cells were included (Figure 7C) to explore if AhRR also mediates apoptosis in non-TNBC breast cancer cells. Whereas, MDA-MB 231 cells were treated for 48 h with the genotoxic drugs, MCF-7 cells were incubated for 72 h. The efficacy of the drugs largely depends on the proliferation rate of the cells. Given that the doubling time of MCF-7 cells (~43 h) is significantly longer than the doubling time of MDA-MB 231 cells (~31 h) as reported (38) we treated the MCF-7 cells 24 h longer than the MDA-MB 231 cells, resulting roughly in the same number of cell divisions before the measurements. Hence, the difference in treatment time may explain that both AhRR-transfected cell-lines exhibit a comparable pro-apoptotic effect. Transient overexpression of rat AhRR or CRISPR/Cas9-mediated AhR knockout resulted in a similarly enhanced susceptibility of both cell lines toward Dox- and EtOP-induced apoptosis (Figures 7A,C). Ectopic overexpression of the rat AhRR has been found to effectively antagonize AhR in human HepG2 and HaCaT cells (39, 40). Overexpression of AhRR in AhR knockout cells yielded a similar level of apoptosis as either condition alone. Collectively, these data suggest that the pro-apoptotic effect of AhRR largely depends on AhR inhibition. As Dox and EtOP induce DNA double-strand breaks (DSB) to initiate apoptosis, we next analyzed the number of cells positive for phosphorylated histone 2AX (γH2AX), an established marker for DSB (41). In fact, AhR deficiency as well as AhRR overexpression resulted in an accumulation of these DNA lesions in both breast cancer cell-lines (Figures 7B,D), suggesting a regulatory function of AhR in DSB repair. Western Blot data and mRNA expression analysis confirm the successful knockout of AhR in MCF-7 and MDA-MB 231 breast cancer cell lines (Figures 7E,F).

## Discussion

**Figure 8:**
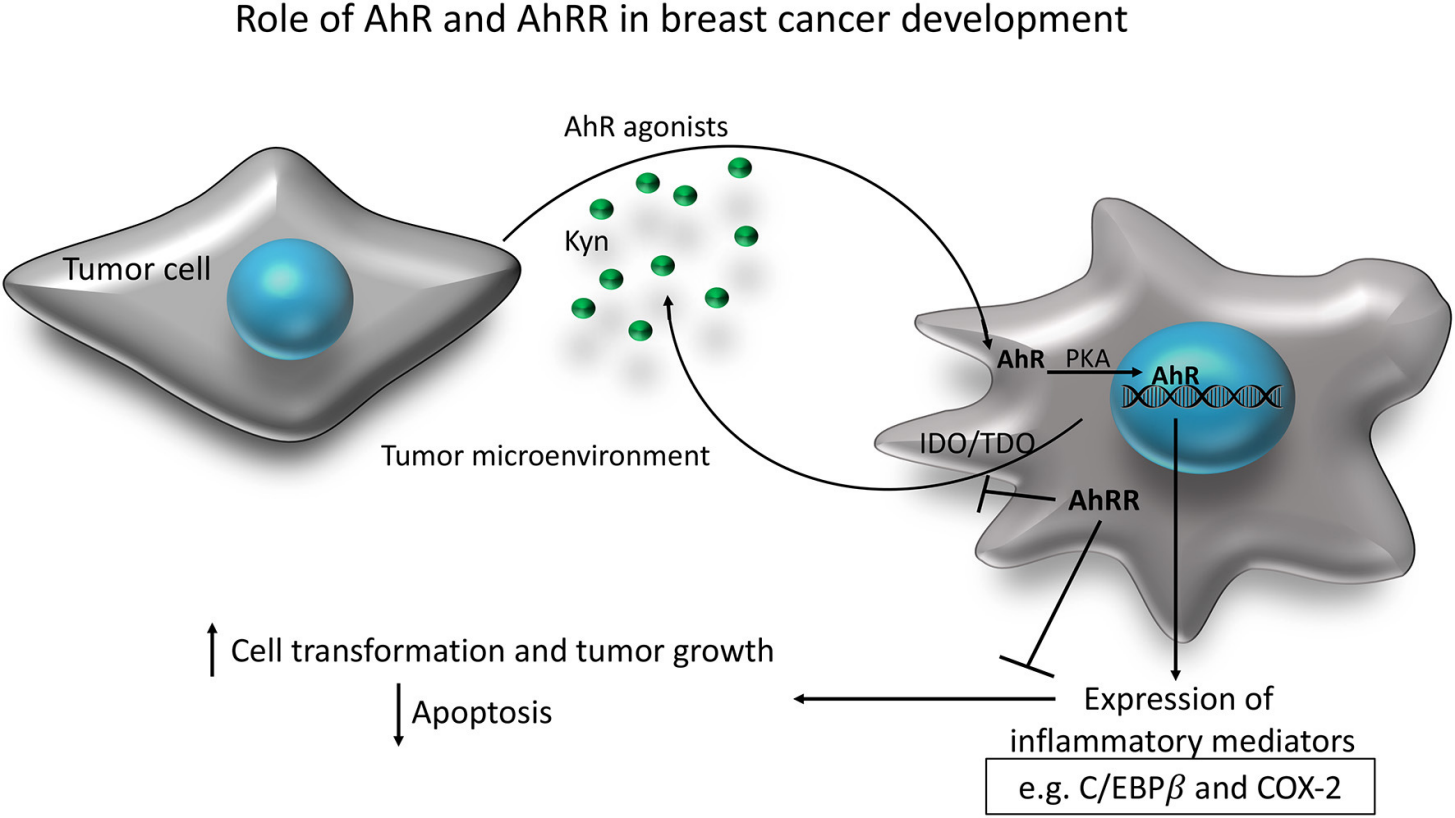
A hypothetical scheme of the role of AhR and AhRR in breast cancer development. AhR agonists like kynurenine (Kyn) are produced in the tumor microenvironment and activate AhR in cells of the tumor microenvironment including stromal and inflammatory cells or the tumor cells themselves. Activation of the PKA pathway and the increased expression of inflammatory mediators such as C/EBPβ and COX-2 contribute to mammary cell transformation and increased cell proliferation. The activated AhR pathway can also induce the activity of the immune-regulatory enzymes IDO and TDO, which metabolize tryptophan into the endogenous AhR ligand Kyn in a positive feedback loop. AhRR may function as an inhibitor of the tumor promoting mechanisms triggered by AhR in the tumor microenvironment. IDO, indoleamine 2,3-dioxygenase; TDO, tryptophan-2,3-dioxygenase; PKA, protein kinase A; Kyn, kynurenine.

In prior work, we demonstrated that overexpression of AhRR *in vitro*, in human breast cancer cells, inhibits cell survival mediated by AhR (17). This study is the first to demonstrate that AhRR overexpression restricts mammary tumor cell growth and tumorigenesis *in vivo*. In the syngeneic E0771 model, we demonstrate that AhRR overexpression inhibits basal and AhR-driven (TCDD-stimulated) mammary tumor cell growth. The results suggest that AhRR overexpression in the host environment is sufficient to inhibit orthotopic growth of mammary tumor cells. This builds on our prior study in which we demonstrated that growth of lymphoma cells was suppressed in AhRR Tg mice (42). We note that the growth of E0771 cells was suppressed in untreated AhRR transgenic mice suggesting that AhRR overexpression in the host may suppress tumor growth independent of exogenous and toxic AhR ligands. Interestingly, a recent report using immortalized mouse mammary gland fibroblasts showed that knockout of AhR also reduced the potential to induce tumors in a mouse xenograft model (43) indicating that suppression of AhR by AhRR as well as deficiency of AhR impairs tumorigenicity. One possibility is that the increased expression and activity of AhR found in breast tumor cells causes altered levels of tryptophan metabolizing enzymes as shown for indoleamine 2,3-dioxygenase (IDO) and tryptophan-2,3-dioxygenase (TDO) (34, 44, 45) and generate high levels of the endogenous AhR ligand kynurenine (Kyn). C/EBPβ as well as COX-2 have recently been found to maintain the constitutive expression of Kyn-producing TDO in human glioblastoma (46, 47), suggesting that this transcription factor and COX-2 critically shape the pro-tumorigenic properties of AhR. The IDO-Kyn-AhR signaling pathway has been shown to mediate immunosuppression involving Tregs and tumor-associated macrophages, which can be reversed by AhR inhibition (48). Interestingly, a recent meta-analysis across military and civilian cohorts indicates that lower AhRR methylation correlates with lower levels of Kyn (49) suggesting that higher AhRR activity may regulate the level of Kyn. Consequently, the IDO-Kyn-AhR signaling pathway provides a new target in cancer immunotherapy as discussed recently (50) and AhRR may provide an important tool to inhibit this pathway (Figure 8).

The PyMT mammary tumorigenesis model is a well-characterized and widely used model of ER-negative, metastatic breast cancer. We find that expression changes in the AhR/AhRR axis observed in human breast cancer (9) are reflected in this model, with overexpression of AhR and its canonical targets COX-2 and C/EBPβ along with down-regulation of AhRR. Notably, we demonstrate that AhRR overexpression in the PyMT background increases tumor latency and decreases tumor incidence and burden. While lung metastases are prevalent in PyMT/wt mice, as expected, we found lung metastases in only 40% of PyMT/AhRR^+^ mice at necropsy. This may be an outcome of decreased primary tumor burden in PyMT/AhRR^+^ mice and/or may reflect a decrease in functional metastatic capacity in PyMT/AhRR^+^ tumor cells. Previous studies have found that AhRR silencing increases tumor cell migration and invasion (8). Further, breast cancer patients who retain high AhRR expression show prolonged metastasis-free survival (9), strongly suggesting that AhRR plays a functional role in limiting biological behaviors which contribute to metastasis.

Further, we tested whether AhRR affects the expression of COX-2 and C/EBPβ as well as the growth of tumor cells *in vitro* using UCD-PYMT, a tumor cell line isolated from a PyMT mammary tumor. Activation of AhR by TCDD of these cells led to an increase in both COX-2 and C/EBPβ, with significant inhibition of this response by AhRR overexpression. Moreover, the constitutive and TCDD-stimulated AhR-mediated DRE reporter activity as well as C/EBPβ DNA binding was abrogated by AhRR overexpression in UCD-PYMT cells, which was associated with the inhibition of cell proliferation. The results are in line with the *in vivo* findings and confirm previous reports showing a reduced cell proliferation, increased apoptosis and inhibition of inflammatory invasion and migration of breast cancer cells by AhRR overexpression (1, 8, 17, 37). Our previous studies found that C/EBPβ and COX-2 are important mediators of an AhR-dependent and TCDD-induced resistance to apoptosis in lymphoma cells, demonstrating their critical role in AhR-driven tumor cell survival (42, 51). In addition to a host-dependent tumor suppressive effect of AhRR indicated by current data from a mouse xenograft model, the results with mammary tumor cells suggest that AhRR mediates also cell-intrinsic responses associated with the suppression of C/EBPβ and COX-2. COX-2 is an inducible isoform upregulated in many cancers (52). In earlier studies, we demonstrated that activation of C/EBPβ drives AhR-mediated COX-2 gene induction *via* activation of PKA (35, 36). Therefore, it is not unlikely that intrinsic- as well as host-dependent effects of AhRR are mediated through repression of the PKA/C/EBPβ pathway causing inhibition of tumor growth. PKA has been found to control cell growth in many cancer types *in vivo* and *in vitro* and represents a potential target for pharmacological treatment of tumors (53). Downstream of PKA, phosphorylation of Src has been shown to initiate mammary cell transformation associated with increased cell proliferation (54). High Src expression has also been defined with basal-like and HER2 human breast cancer associated with poor clinical outcome (55). Notably, induction of COX-2 by AhR is mediated through a mechanism involving rapid activation of Src kinase and PKA by AhR (35, 36, 56).

Interestingly, the selective inhibition of COX-2 has been shown to significantly increase apoptosis in tumors and to decrease the number and size of tumors in the PyMT mouse model (57, 58). Degner et al. (59) have shown that AhR ligands can upregulate COX-2 expression, which led to a pro-inflammatory local environment that supported tumor development. The generation of inflammatory mediators are a critical component of the tumor microenvironment and tumorigenesis (60). Furthermore, elevated expression levels of C/EBPβ have been associated with the progression of breast and ovarian cancers and are correlated with an unfavorable prognosis (61–63). This is supported by studies on different mouse models for metastatic breast cancer, showing that C/EBPβ induces the expression of genes relevant for metastasis to the lungs (63, 64). Interestingly, Wiegmans et al. reported that C/EBPβ cooperates with RAD51, a key protein of homologous recombination repair, to control invasion- and metastasis-associated gene expression (64).

Resistance to apoptosis and chemotherapy is a major factor driving breast cancer mortality, particularly in TNBC where targeted therapies are not available for most patients. We report here that AhRR overexpression sensitizes PyMT-derived mammary tumor cells and human breast cancer cells to both Dox and EtOP. AhRR overexpression is as effective as AhR deletion, suggesting that AhRR restoration is a feasible approach for addressing chemoresistance. The elevated levels of γH2AX observed in AhR-compromised breast cancer cells exposed to genotoxic drugs, supports previous studies showing that AhR plays an important role in repair of DSB (40, 65, 66). In several malignancies, including a proportion of triple negative breast cancers, elevated DSB repair activities impair therapeutic efficacy by enhancing the resistance toward therapeutically induced DNA damage (67, 68). Interestingly, C/EBPβ was shown to protect ovarian cancers against cisplatin treatment by enforcing the expression of genes involved in drug transport, cell survival, and DNA repair, more precisely homologous recombination repair and non-homologous end-joining (61). However, to what extent AhR's impact on DSB repair and apoptosis depends on C/EBPβ is not well-understood and currently under investigation.

In summary our results demonstrate that AhRR overexpression suppresses mammary tumor growth and progression and is associated with the repression of markers of inflammation and tumor cell survival, particularly if the AhR is constantly activated by endogenous or persistent toxic environmental ligands. The AhRR may suppress extrinsic and tumor cell intrinsic oncogenic pathways in the tumor microenvironment to protect from chronic inflammation and tumorigenesis (Figure 8). It is important to note that AhR signaling in mammary microenvironment cells has been observed previously. For example, growth of mouse mammary fibroblasts as leimyosarcomas *in vivo* was decreased by AhR deletion (43). In addition, human breast cancer-associated fibroblasts upregulate the canonical AhR target gene, CYP1B1, and proliferate in response to treatment with the AhR ligand, 3-methylcholanthrene (69). While our results suggest that AhRR overexpression in the host environment is sufficient to decrease mammary tumor cell growth, the tumor cell-intrinsic vs. extrinsic roles of AhR/AhRR are complex and have to be explored in more detail. Additional studies are needed and will address approaches to functionally restore AhRR in AhRR silenced tumors.

## Data Availability Statement

The original contributions presented in the study are included in the article/Supplementary Material, further inquiries can be directed to the corresponding author.

## Ethics Statement

The animal study was reviewed and approved by Institutional Animal Care and Use Committee (IACUC).

## Author Contributions

Project was planned by CFAV and CS. Plasmids, cell lines, and critical resources were provided by ML, AR, AB, JJ, HM and YI. CFAV, GL, SK, YH, CD, AC, YI, CS, CV, and ML performed the majority of the experiments and interpreted the data. Statistical analyses were performed by SK, CV, and YH. Original draft preparation was done by CFAV, TH-S, and CS. All authors reviewed the manuscript.

## Conflict of Interest

The authors declare that the research was conducted in the absence of any commercial or financial relationships that could be construed as a potential conflict of interest.
