# Supplementary material for: Targeting the Aryl Hydrocarbon Receptor Signaling Pathway in Breast Cancer Development
Source: Front Immunol. 2021 Mar 8;12:625346. doi: 10.3389/fimmu.2021.625346 (PMC7982668; doi:10.3389/fimmu.2021.625346)
Supplement: Supplementary file 1 [file Image_1.pdf]

Supplemental Figure 1

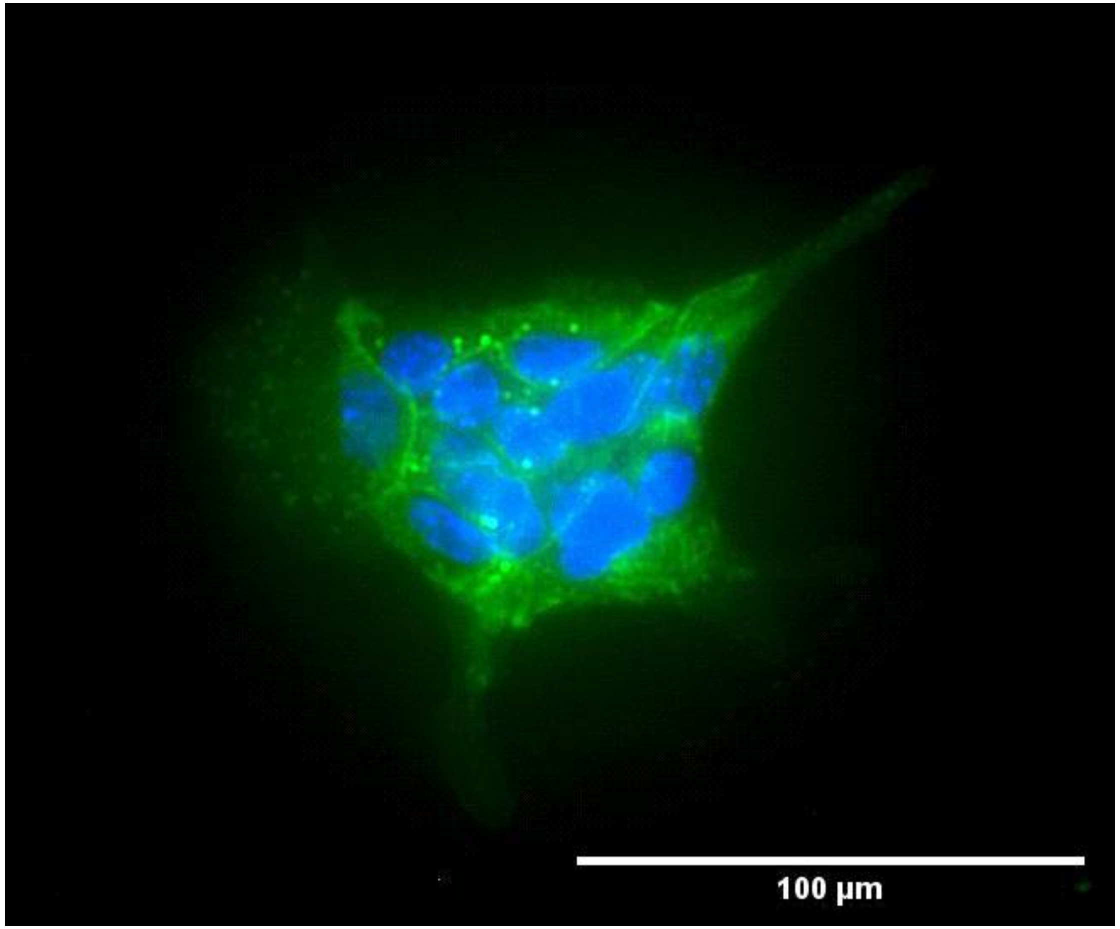

Supplemental Figure 1. Expression of epithelial cell-specific marker E-Cadherin is detected in UCD-PYMT cells. UCD-PYMT cell colonies, defined by the interaction of at least 2 cells, were stained with anti-E-Cadherin antibody. E-Cadherin expression (green) was detected in all UCD-PYMT cell colonies. Image is representative of median E-Cadherin fluorescecnce intensity in colonies cultured to day 5. Slides were costained with DAPI (blue) to visualize nuclei. Original magnification 40x.
